# Diagnostic performance of the Minimal Eating Observation and Nutrition Form - Version II (MEONF-II) and Nutritional Risk Screening 2002 (NRS 2002) among hospital inpatients - a cross-sectional study

**DOI:** 10.1186/1472-6955-10-24

**Published:** 2011-12-20

**Authors:** Albert Westergren, Erika Norberg, Peter Hagell

**Affiliations:** 1 The PRO-CARE group, School of Health and Society, Kristianstad University, Kristianstad, Sweden; 2 Central Hospital, Kristianstad, Sweden

## Abstract

**Background:**

The usefulness of the nutritional screening tool Minimal Eating Observation and Nutrition Form - Version II (MEONF-II) relative to Nutritional Risk Screening 2002 (NRS 2002) remains untested. Here we attempted to fill this gap by testing the diagnostic performance and user-friendliness of the MEONF-II and the NRS 2002 in relation to the Mini Nutritional Assessment (MNA) among hospital inpatients.

**Methods:**

Eighty seven hospital inpatients were assessed for nutritional status with the 18-item MNA (considered as the gold standard), and screened with the NRS 2002 and the MEONF-II.

**Results:**

The MEONF-II sensitivity (0.61), specificity (0.79), and accuracy (0.68) were acceptable. The corresponding figures for NRS 2002 were 0.37, 0.82 and 0.55, respectively. MEONF-II and NRS 2002 took five minutes each to complete. Assessors considered MEONF-II instructions and items to be easy to understand and complete (96-99%), and the items to be relevant (87%). For NRS 2002, the corresponding figures were 75-93% and 79%, respectively.

**Conclusions:**

The MEONF-II is an easy to use, relatively quick and sensitive screening tool to assess risk of undernutrition among hospital inpatients. With respect to user-friendliness and sensitivity the MEONF-II seems to perform better than the NRS 2002, although larger studies are needed for firm conclusions. The different scoring systems for undernutrition appear to identify overlapping but not identical patient groups. A potential limitation with the study is that the MNA was used as gold standard among patients younger than 65 years.

## Background

Undernutrition is associated with poorer health, compromised ability to recover from medical conditions and increased mortality [1]. People at risk for or with manifest undernutrition therefore need to be identified in order to initiate prevention or interventions. Low Body Mass Index (BMI) and unintentional weight loss are considered key indicators of undernutrition [2], and together with change in food intake these indicators are associated with changes in function and clinical outcome [3]. This is reflected in commonly used nutritional screening tools such as the Nutritional Risk Screening 2002 (NRS 2002; [4]), the Mini Nutritional Assessment (MNA; [5,6] ), Malnutrition Universal Screening Tool (MUST; [7]) and the recently developed Minimal Eating Observation and Nutrition Form - Version II (MEONF-II; [8,9]).

In Sweden, it is recommended that undernutrition risk screening should include at least the following three criteria: unintentional weight loss, eating difficulties, and low BMI [10]. These criteria were recently operationalized in the MEONF-I [11] and its subsequent modification MEONF-II [8,9]. While the NRS 2002 and MNA have been widely used for years, the MEONF-II was only recently introduced [8,9].

MEONF-II is based within an interdisciplinary nursing framework, combining descriptions of mealtime problems with classical signs of undernutrition in order to facilitate detection of problems in need of interventions [8,9]. In a previous study, the sensitivity, specificity and user-friendliness of the MEONF-II and MUST in relation to the MNA was analyzed among elderly (>65 years) orthopaedic, cardiology and stroke inpatients. The MEONF-II was found to be easy and relatively quick to use with a sensitivity of 0.68, which was favourable to that of the MUST (0.57) [8]. However, its usefulness relative to the NRS 2002 remains untested. Here we attempted to fill this gap by testing the MEONF-II and NRS 2002 in relation to the MNA among hospital inpatients.

## Methods

### Sample

Ninety six persons (18+ years old) receiving inpatient care at four wards (stroke, surgery, orthopaedic, internal medicine) at a hospital in southern Sweden were approached. Fifteen of the patients were younger than 65 years. There were no predefined inclusion or exclusion criteria used. Data from 87 patients were analyzed. Reasons for not participating (n = 7) were aphasia (n = 1), language difficulties (n = 1), dementia (n = 1), mental illness (n = 1), and not wanting to participate (n = 3). In another two cases the forms were incomplete and had to be excluded. Informed consent was obtained. The sample has previously been used as part of a larger study regarding MEONF-II cut-off scores [9]. The study was approved by the local ethics council at Kristianstad University.

### Assessments

Background data (i.e. age, sex, admitted to hospital from own home/special accommodation, and cohabitation) were recorded and assessments were conducted according to the MNA, MEONF-II and NRS 2002.

#### Mini Nutritional Assessment (MNA)

The MNA was developed for use among elderly patients (≥ 65 years) [12,13]. The full MNA consists of 18 items with a maximum possible total score of 30. The cut-off values for MNA have been defined based on serum albumin levels [13,14]. A score below 17 is indicative of undernutrition, patients scoring 17 to 23.5 are at risk for undernutrition, and patients with a score of 24 or more are considered well-nourished [13,14]. The tool has been shown to have high sensitivity (96%), specificity (98%), and positive predictive value (97%) when compared with extensive assessments of patients' nutritional status [6]. Here we used the 18-item MNA as the gold standard for determination of nutritional status.

#### Nutritional Risk Screening 2002 (NRS 2002)

The main purpose of the NRS 2002 is to identify patients who may benefit from nutritional interventions [4]. It consists of four initial screening questions: 1) is BMI < 20.5?; 2) has intake been reduced during the last week?; 3) has there been a recent weight loss?; 4) is the patient severely ill? Patients without any affirmative responses to the four initial screening questions were classified as "not at nutritional risk". A positive response to ≥1 of these four questions prompts a formal screening that is characterized by scoring two components, undernutrition and severity of disease. The undernutrition component comprises BMI, percent recent weight loss and recent change in food intake; the disease severity component considers increase in nutritional requirements resulting from disease, i.e. stress-metabolism. A score of 0-3 (representing absent, mild, moderate and severe) is assigned to each of the two components. In addition, a score of one is added for people ≥70 years old. Thus, the final score can range from 0-7. A score of 3 or above is considered signalling nutritional risk. The cut-off value for NRS 2002 has been identified by a retrospective classification, and application of cut-off scores in randomized controlled trials identifying the effect of nutritional intervention (typically artificial nutrition) on clinical outcome as positive or absent, and by using receiver operating characteristic curve (ROC analysis) [4]. In a previous study among geriatric (>65 years) hospital patients, the NRS 2002 had a sensitivity of 0.39, specificity of 0.83, positive and negative predictive values of 0.84 and 0.37, respectively, and an accuracy of 0.52 when compared with the MNA [15].

#### Minimal Eating Observation and Nutrition Form - Version II (MEONF-II)

MEONF (additional file 1) was developed from the Minimal Eating Observation Form - Version II (MEOF-II) [16,17] and the criteria unintentional weight loss, either low BMI (< 20 for 69 years or younger, or < 22 for 70 years or older) [10] or calf circumference < 31 centimeters, and an additional assessment of the presence or absence of clinical signs of undernutrition [8]. MEOF-II includes three components of eating. *Food intake *includes "difficulty manipulating food on plate", "difficulty conveying food to the mouth" and "difficulty to maintain good sitting position during meals". *Swallowing/mouth *includes "difficulty chewing", "difficulty coping with food in mouth" and "difficulty swallowing". *Energy/appetite *includes "lacks energy to complete an entire meal", "poor appetite" and "eats less than 3/4 of food served" [16]. In MEONF-II all items are scored 1 except for unintentional weight loss and energy/appetite, which are scored 2 since such problems are significant indicators or predictors of undernutrition [2,16,17]. MEONF-II yields a total score ranging from 0-8. A score of 0-2 is interpreted as low risk for undernutrition, a score of 3-4 is considered a moderate risk, and a score ≥5 as high risk for undernutrition [8]. These cut-off scores were initially based on clinical reasoning [8], and later confirmed by using ROC analysis comparing MEONF-II scores against the MNA classification [9]. Among patients older than 65 years, MEONF-II has shown a sensitivity of 0.73, specificity 0.88, positive predictive value 0.81, negative predictive value 0.82, and accuracy of 0.82 when compared with the MNA [8].

### Procedure

Patients were assessed during the first four days after admission. Data were collected by one nurse on each ward (each having special responsibility for nutrition at their respective wards) during one set day in November 2010.

The order of the nutritional assessments in the protocol was, first MEONF-II, thereafter NRS 2002, and finally MNA. Meal-time observations (MEONF-II) were conducted during lunch or dinner. Height and weight were recorded in the morning and other observations at convenient time points during the day. The four nurses received written and oral information about the study and the included assessment methods. The information was provided to the four nurses in a single group session lasting for about one hour the day before data collection. The researchers were available to answer questions during the data collection day.

User-friendliness of the three tools was evaluated by recording the time required to complete each tool and by inquiring the assessors of their perceived ease of understanding and following instructions, ease of understanding and completing items, and whether items were perceived as relevant. This was done following each patient assessment (n = 87).

### Analyses

The diagnostic performance of the MEONF-II and NRS 2002 was assessed by calculating the sensitivity, specificity, positive and negative predictive values (PPV and NPV, respectively) and accuracy [18,19], using results from the 18-item MNA as the comparator gold standard. These indices provide values ranging from zero to one (or, equivalently expressed as a percentage), where higher values are preferred [18,19]. For these analyses, patients with undernutrition and those at risk for undernutrition according to the MNA were collapsed into one group. Similarly, patients with moderate and high risk for undernutrition according to the MEONF-II were collapsed into one group. 95% confidence intervals (CIs) were constructed for sensitivity, specificity, PPV and NPV; non-overlapping 95% CIs between NRS 2002 and MEONF-II was regarded statistically significant. Since the MNA was developed for people >65 years of age, data were also analyzed with people younger than 65 years excluded (n = 15).

Time to complete the three screening tools was analyzed by Friedman's two-way analysis of variance by ranks followed by post-hoc Wilcoxon signed ranks tests. Other user-friendliness data, and undernutrition risk according to the three tools were analyzed using Cochran's Q test followed by post-hoc analyses (McNemar). P-values were considered significant if < 0.05 (following Bonferroni corrections in post-hoc analyses)[18]. Analyses were conducted using PASW Statistics 18.0.

## Results

Demographical data are presented in Table 1.

**Table 1:** Demographic data

	Ward				
		
	Stroke	Surgery	Orthopaedic	Geriatric Medicine	Total
	n = 21	n = 19	n = 23	n = 24	n = 87
**Age**					
mean (SD)	72.1 (18.1)	69.6 (13.7)	75.0 (15.8)	82.6 (8.6)	74.8 (15.1)
min-max	23-91	41-88	37-92	58-98	23-98
**Sex**					
Women, n (%)	11 (52)	8 (42)	12 (52)	17 (71)	48 (55)
Men, n (%)	10 (48)	11 (58)	11 (48)	7 (29)	39 (45)
**Admitted to hospital from**^1^					
Ordinary/own home, n (%)	20 (95)	19 (100)	20 (87)	22 (96)	81 (94)
Special accomodation, n (%)	1 (5)	0	3 (13)	1 (4)	5 (6)
**Cohabitation**^**2**^					
Married/living with someone, n (%)	10 (48)	10 (56)	9 (45)	8 (35)	37 (45)
Alone, n (%)	11 (52)	8 (44)	11 (55)	15 (65)	45 (55)

^1) ^Internal attrition/drop-out n = 2

^2) ^Internal attrition/drop-out n = 5

The proportion of people classified as at risk for undernutrition according to NRS 2002 (29%) was significantly lower than found using MEONF-II (45% at moderate/high risk, p = 0.007) and MNA (60% at risk for undernutrition/undernourished, p < 0.0005). There was no significant difference (p = 0.021) in proportions found using the MEONF-II and MNA following Bonferroni correction (p = 0.063) (Table 2). Out of 18 undernourished patients according to MNA 13 were considered being at high risk according to MEONF-II, and 12 as at risk for undernutrition according to NRS 2002. Out of 22 patients at high risk according to MEONF-II, 13 were considered undernourished according to MNA, and 12 as at risk according to NRS 2002.

**Table 2:** Percentage of individuals classified as at risk of undernutrition (UN)

	Ward				
		
	Stroke	Surgery	Orthopaedic	Geriatric Medicine	Total
	n = 21	n = 19	n = 23	n = 24	n = 87
**MEONF-II**, n (%)					
Low risk for UN (0-2 points)	11 (52)	11 (58)	12 (52)	14 (58)	48 (55)
Moderate risk for UN (3-4 points)	5 (24)	3 (16)	3 (13)	6 (25)	17 (20)
High risk for UN (≥ 5 points)	5 (24)	5 (26)	8 (35)	4 (17)	22 (25)
					
**NRS 2002**, n (%)^1^					
No risk for UN (≤ 3 points)	15 (71)	16 (84)	16 (73)	14 (58)	61 (71)
Risk for UN (≥ 3 points)	6 (29)	3 (16)	6 (27)	10 (42)	25 (29)
					
**MNA**, n (%)^2^					
Well nourished (≥2 4 points)	8 (38)	5 (26)	11 (52)	10 (42)	34 (40)
Risk for UN (17-23.5 points)	8 (38)	10 (53)	3 (14)	12 (50)	33 (39)
UN (≤ 17 points)	5 (24)	4 (21)	7 (33)	2 (8)	18 (21)

^1) ^Internal attrition/drop-out n = 1 (orthopaedic ward), ^2) ^Internal attrition/drop-out n = 2 (orthopaedic ward)

MEONF-II, Minimal Eating Observation and Nutrition Form - Version II; MNA, Mini Nutritional Assessment; NRS 2002, Nutritional Risk Screening 2002.

The sensitivity (i.e., proportion of people correctly identified as at risk for undernutrition according to the 18-item MNA) of the MEONF-II was 61% (Table 3). For the NRS 2002, sensitivity was 37%. That is, the two methods missed 39% and 63%, respectively, of cases identified as at risk for or being undernourished according to the MNA. The specificity (i.e., proportion of people correctly identified as not at risk for undernutrition according to the 18-item MNA) for the MEONF-II was 79%, and for the NRS 2002 it was 82% (Table 3).

**Table 3:** Diagnostic performance of the MEONF-II and NRS 2002 compared to the 18-item MNA (n = 85)

	**Number of patients**^**1**^								
	A	B	C	D	**SENS **^**2**^ (95% CI)	**SPEC **^**3**^ (95% CI)	**PPV **^**4**^ (95% CI)	**NPV **^**5**^ (95% CI)	**Accuracy **^**6**^
MEONF-II in relation to MNA	31	7	20	27	.61 (.46-.74)	.79 (.62-.91)	.82 (.66-.92)	.57 (.42-.72)	.68
NRS 2002 in relation to MNA	19	6	32	28	.37 (.24-.52)	.82 (.65-.93)	.76 (.55-.91)	.47 (.34-.60)	.55

	MNA								
Screening (MEONF-II or NRS 2002)	UN-risk/UN		Not at risk						
UN-risk/UN	A		B						
Not at risk	C		D						

^1) ^Internal attrition/drop-out n = 2 (orthopaedic ward) ^2) ^SENSitivity = A/(A+C); ^3) ^SPECificity = D/(B+D); ^4) ^Positive Predictive Value (PPV) = A/(A+B); ^5) ^Negative Predictive Value (NPV) = D/(C+D); ^6) ^Accuracy = A+D/(A+B+C+D)

MNA, Mini Nutritional Assessment; CI, confidence interval; MEONF-II, Minimal Eating Observation and Nutrition Form - Version II; NRS 2002, Nutrition Risk Screening 2002; UN, Undernutrition.

A positive MEONF-II result, indicating risk for undernutrition, was associated with a PPV of 82%; that is, a 82% probability that the individual really was undernourished (according to the 18-item MNA). A negative MEONF-II result was associated with a NPV of 57%; that is, a 57% probability that the individual really was not undernourished. For the NRS 2002, PPV and NPV were 76% and 47%, respectively. The exact proportions of agreement (accuracies) according to the various methods were 68% for MEONF-II and 55% for NRS 2002 in relation to the 18-item MNA (Table 3).

Overlapping 95% CIs between the NRS 2002 and MEONF-II suggested no statistically significant differences in sensitivity, specificity, PPV and NPV between these tools.

Analyses of data only from those ≥65 years old (n = 72) yielded similar results for both the MEONF-II (sensitivity, 57%; specificity, 79%; PPV, 80%; NPV, 56%; accuracy, 66%) and NRS 2002 (sensitivity, 36%; specificity, 79%; PPV, 71%; NPV, 46%; accuracy, 53%).

The median time required to conduct assessments according to the NRS 2002 and MEONF-II was 5 minutes each; in contrast, the 18-item MNA took twice as long to complete (Table 4). Assessors considered the MEONF-II instructions and items easy to understand and complete (96-99%), and its items to be relevant (87%). For the NRS 2002, the corresponding figures were 75-93% and 79%, respectively. Both the NRS 2002 and MEONF-II were significantly quicker to complete than the MNA (Table 4). Items of the MNA and MEONF-II were considered easier to understand compared to NRS 2002 items, and MEONF-II items were considered easier to answer than NRS 2002 items (Table 4).

**Table 4:** User-friendliness of the MNA, MEONF-II and NRS 2002, n = 87

	MNA	NRS 2002	MEONF-II	P-value ^1^
Time to complete, minutes median	10	5	5	< 0.0005 ^2^
q1-q3	8-10	2-10	2-10	
min-max	3-20	1-20	1-20	
Instructions easy to understand, %	93	93	99	0.165
Items easy to understand, %	93	81	97	< 0.0005 ^3^
Items easy to answer, %	85	75	96	< 0.0005 ^4^
Items relevant, %	78	79	87	0.247

^1) ^Friedman's two-way analysis of variance by ranks followed by post-hoc Wilcoxon signed ranks tests (time to complete), and Cochran's Q test followed by post-hoc McNemar tests (other data).

^2) ^Significant difference between MNA and NRS 2002, MNA and MEONF-II (both comparisons p = 0.001; p = 0.003 following Bonferroni correction).

^3) ^Significant difference between MNA and NRS 2002 (p = 0.004; p = 0.012 following Bonferroni correction), and between NRS 2002 and MEONF-II (p = 0.001; p = 0.003 following Bonferroni correction).

^4) ^Significant difference between NRS 2002 and MEONF-II (p = 0.001; p = 0.003 following Bonferroni correction).

MNA, Mini Nutritional Assessment; MEONF-II, Minimal Eating Observation and Nutrition Form - Version II; NRS 2002, Nutrition Risk Screening 2002; q1-q3, inter-quartile range (25^th^-75^th ^percentile).

## Discussion

This study provides support for the validity and user-friendliness of the MEONF-II and the NRS 2002. Perceived user-friendliness of the MEONF-II was somewhat better than that of the NRS 2002. It was indicated, although not significantly, that the MEONF-II had better sensitivity than the NRS 2002 in comparison to the MNA.

The MNA has commonly been used as the comparator, or gold standard nutritional screening tool (e.g., [7-9,15,20,21]). MNA captures patients at risk also in an early stage so that preventive actions can be taken [12]. The NRS 2002 differs somewhat in focus in that its goal is to identify patients that are most likely to benefit from nutritional interventions [4,15]. It is therefore possible that the NRS 2002 is less suited for detecting patients in need for preventive actions than the MNA is. The intention of the MEONF-II, on the other hand is both to identify patients needing preventive nutritional interventions and those needing nutritional treatment. Furthermore, the MNA was developed for people ≥65 years [12,13], and the use of it as gold standard among younger patients can be questioned. However, we did not find any relevant differences between results from analyses of the full sample and when excluding the younger subsample. Thus, whether the MNA can be considered an appropriate gold standard or not depends on the intention with the comparator and possibly the age of the patients in the sample. In any case, people at risk for undernutrition need further assessments and no instrument can alone capture all aspects influencing the eating situation and the nutritional intake.

Cut-off scores used in the different instruments affect the results. The MNA cut-offs have been defined based on serum albumin values, a predictor of morbidity and mortality in elderly people [13,14]. This would suggest that any comparison with the MNA may only indicate whether low albumin levels can be detected. However, the MNA has also been validated against more extensive assessments of nutritional status (including, e.g., additional biomarkers and dietary parameters) [6]. The cut-off scores for MEONF-II have been defined based on clinical reasoning and confirmed by ROC analysis against the MNA classification [8,9], and the NRS 2002 cut-off was based on findings from randomized controlled trials regarding the effect of nutritional intervention [4]. However, the classification of patients in that study was done retrospectively and the authors were not blinded to outcome (usually artificial nutrition) when estimating the degree of undernutrition and severity of disease [4]. The different intentions of these instruments, the way they have been developed and compared with other measures affect the prevalence findings. For instance, in this study the NRS 2002 identified a significantly lower percentage (29%) of patients as at risk than the MEONF-II (45%) and MNA did (60%). Similarly, in another study [15] the MNA identified 70% of patients as at risk or malnourished while the NRS 2002 identified 40%. It therefore appears that the MEONF-II does not identify patients at risk as early as the MNA, and not as late as the NRS 2002. In clinical practice such differences will have consequences for preventive and treatment actions. Further on, a majority of those being undernourished according to MNA were correctly classified as at high risk by MEONF-II (13 out of 18 patients) or at risk by NRS 2002 (12 out of 18 patients). Anyhow, it should be remembered that the main purpose with screening is to identify people at risk and not to decide whether it is a low or high risk and that any case being at risk needs a more detailed assessment. In addition, efforts are needed to develop a clear vocabulary and uniform definitions of risk (low/high) and manifest undernutrition.

The MEONF-II showed a 68% concordance with the MNA, which is lower than that observed in a previous study (82%) [8]. One explanation to the difference in accuracy could be that in the previous study [8], the assessment procedures were reviewed individually with the nurse assessors, whereas it was conducted as a group session in this study. However, the accuracy and sensitivity of the NRS 2002 found here (55% and 37%) are similar to those in previous studies of this instrument in relation to the MNA (52% and 39%) [15].

The MEONF-II is a screening tool designed to detect risk of undernutrition, not only those with manifest undernutrition. As such, it is reasonable for sensitivity to be given priority at the cost of specificity since over-identification is preferable to under-identification, given that positive screening results are followed by in-depth assessment [19,22]. In this respect the NRS 2002 appears less well suited, since its sensitivity was lower (37%) compared to that of MEONF-II (61%). However, as the associated 95% CIs overlapped, additional studies in larger samples are needed before any firm conclusions can be drawn.

The MEONF-II demonstrated good user-friendliness in terms of time to complete, ease of understanding of items, as well as ease of completion. In these respects, our observations suggest that MEONF-II compares favourably to the NRS 2002. One reason for this may be that it helps nurses identifying problems and intervene directly, either themselves or by involving other professionals. It should be noticed that time consumption was low when using MEONF-II, despite the fact that this assessment was done before NRS 2002 and MNA. It could otherwise be expected that the time needed would be lower for tools used as second and third since several items are shared between the tools. One should, however, be careful in the interpretation of these findings since it was only four nurses that rated user-friendliness and there may be a learning curve for each of these nurses, affecting rating of user-friendliness and time needed for completing forms. Anyhow, user-friendliness is of fundamental importance for successful clinical implementation of nutritional screening tools. As most screenings are carried out by nurses, their perspective in this respect must be taken into account.

## Conclusion

The MEONF-II is an easy to use, relatively quick and sensitive screening tool to assess risk of undernutrition among hospital inpatients. High sensitivity is of primary concern in nutritional screening. With respect to user- friendliness and sensitivity, the MEONF-II appears to perform well compared to the NRS 2002, although larger studies are needed for firm conclusions. The different scoring systems for undernutrition appear to identify overlapping but not identical patient groups. However, the appropriateness of using the MNA as gold standard among patients younger than 65 years can be questioned.

## Competing interests

The authors declare that they have no competing interests.

## Authors' contributions

All authors participated in the design of the study, read and approved the final manuscript. AW informed the data collectors, performed the statistical analysis, and drafted the manuscript. EN coordinated and supervised the data collectors. PH helped to draft the final manuscript.

## Pre-publication history

The pre-publication history for this paper can be accessed here:

http://www.biomedcentral.com/1472-6955/10/24/prepub

## Supplementary Material

Additional file 1 **MEONF-II (Minimal Eating Observation and Nutrition Form - Version II)**. The file contains the nutritional screening tool Minimal Eating Observation and Nutrition Form - Version II (MEONF-II). Click here for file
