# Supplementary material for: Diagnostic performance of the Minimal Eating Observation and Nutrition Form - Version II (MEONF-II) and Nutritional Risk Screening 2002 (NRS 2002) among hospital inpatients - a cross-sectional study
Source: BMC Nurs. 2011 Dec 20;10:24. doi: 10.1186/1472-6955-10-24 (PMC3305676; doi:10.1186/1472-6955-10-24)
Supplement: Additional file 1 — MEONF-II (Minimal Eating Observation and Nutrition Form - Version II). The file contains the nutritional screening tool Minimal Eating Observation and Nutrition Form - Version II (MEONF-II). [file 1472-6955-10-24-S1.pdf]

Additional file 1

MEONF-II (Minimal Eating Observation and Nutrition Form – Version II)

			POINTS
1	Unintentional weight loss (regardless of time & magnitude)	Yes, weight loss = 2<br>No weight loss = 0<br>Don't know = leave empty and continue	
2a<br><br><br><br>2b	☐ BMI is less than 20 (69 years or younger)<br>☐ BMI is less than 22 (70 years or older)<br>Height / weight cannot be obtained, measure calf circumference (2b)<br>☐ Calf circumference is less than 31 centimeters	Low BMI or small calf circumference = 1<br>Otherwise = 0	
3	Eating problems (mark with check on left and score according to instructions on right)<br>Food intake<br>☐ Difficult to maintain good sitting position during meals<br>☐ Difficulty manipulating food on plate<br>☐ Difficulty conveying food to mouth	One/more problems = 1<br>No problems = 0	
4	Swallowing/mouth<br>☐ Difficulty chewing<br>☐ Difficulty coping with food in mouth<br>☐ Difficulty swallowing	One/more problems = 1<br>No issues = 0	
5	Energy/appetite<br>☐ Eats less than ¾ of food served<br>☐ Lacks energy to complete an entire meal<br>☐ Poor appetite	One/more problems = 2<br>No problems = 0	
6	Clinical signs indicate risk of undernutrition. Assess e.g., body morphology, subcutaneous fat, muscle mass, grip strength, edema (fluid retention), blood tests (e.g. serum albumin).	Clinical signs indicate risk = 1<br>Otherwise = 0	
	Tally observations 1-6<br>(min = 0, max = 8)	TOTAL:	

RISK OF UNDERNUTRITION<br>☐ 0-2 points, no or low risk<br>☐ 3-4 points, moderate risk<br>☐ 5 points or more, high risk

Gradation of high BMI<br>Overweight:	Obesity:	Severe/morbid obesity:
25-29.9 (69 years or younger)<br>27-31.9 (70 years or older)<br>☐	30-39.9 (69 years or younger)<br>32-41.9 (70 years or older)<br>☐	>40 (69 years or younger)<br>>42 (70 years or older)<br>☐

Westergren et al. BMC Nursing 2011 10:24 doi:10.1186/1472-6955-10-24
